# Zarte Hände bringen Erleichterung. Thure-Brandt-Massage als Zeitdokument und Gründungshilfe für die Urologie

**DOI:** 10.1007/s00120-022-02005-0

**Published:** 2023-01-13

**Authors:** Florian G. Mildenberger, Friedrich H. Moll

**Affiliations:** 1 Institut für Geschichte, Theorie und Ethik der Medizin, Heinrich-Heine-Universität Düsseldorf, Düsseldorf, Deutschland; 2 Museum, Bibliothek und Archiv zur Geschichte der Urologie Düsseldorf-Berlin, Düsseldorf-Berlin, Deutschland; 3 Deutsche Gesellschaft für Urologie e. V., Düsseldorf Berlin, Deutschland; 4 Urologische Klinik, Kliniken der Stadt Köln GmbH, Neufelder Straße 32, 51067 Köln, Deutschland

**Keywords:** Genitalmassage, Abtreibung, Naturheilkunde, Thure Brandt, Masturbation, Psychosomatik, Genital massage, Abortion, Naturopathy, Thure Brandt, Masturbation, Psychosomatic medicine

## Abstract

Naturheilkunde und Urologie berühren sich in der Gegenwart kaum, aber im viktorianischen Zeitalter war es eine Genitalmassage, die der Ärzteschaft verdeutlichte, dass eine auf die Erkrankungen des Unterleibs spezialisierte Ausbildung für Ärzte notwendig war, da ansonsten die Patientinnen Laienheilkundige aufsuchen würden und keine Kliniken. Diese Massage war von dem schwedischen Offizier Thure Brandt in den 1850er-Jahren entwickelt worden. Sie blieb bis nach dem Zweiten Weltkrieg ein Thema in deutschen Arztpraxen.

## Abstract (en)

Naturopathy and urology have little overlap in the present day, but in the Victorian era it was genital massage that made it clear to the medical profession that training specialized in diseases of the abdomen was necessary for physicians, otherwise patients would seek out lay healers and not clinics. This massage was developed in the 1850s by the Swedish officer Thure Brandt. It remained part of German medical practice until after World War II.

Urologie und Naturheilkunde waren und sind keine natürlichen Verbündeten. Es gibt einige Kollegen, die beide Therapierichtungen anbieten, aber blickt man in die Geschichte der Fächer, so scheint es (außerhalb von Kurorten) keine wirklichen Berührungspunkte gegeben zu haben [1]. Allerdings begünstige die erfolgreichste naturheilkundliche Behandlungsmethode für Erkrankungen des Unterleibs des späten 19. Jahrhunderts indirekt die Herausbildung einer modernen Urologie. Denn die ab 1886 aus Schweden importierte „Thure-Brandt-Massage“ zur Behebung von Unterleibserkrankungen bei Frauen (und Männern) verlangte eine detaillierte Kenntnis der harnableitenden Organe, der Komplikationen von Schwangerschaften und die Differentialdiagnose von Neuralgien und Karzinomen [2]. Auch weitere physikalische Therapien aus Schweden besaßen zu Beginn des 20. Jahrhunderts eine weite Verbreitung, wie die so genannten Zander Institute belegen [3]. Darüber hinaus war die Massage von einem Laien entwickelt worden, der in seinen Publikationen die Einfachheit der Anwendung betonte und ausdrücklich Frauen ermunterte, medizinisch tätig zu werden, obwohl diesen damals in Deutschland das Universitätsstudium, nicht aber die laienheilkundliche Tätigkeit noch untersagt war. In anderen Worten: es bedurfte der Herausbildung einer neuen ärztlichen Fachrichtung, um die Unterleibsmassage zu verwissenschaftlichen und das ärztliche Behandlungsmonopol zu sichern.

## Vorgeschichte

Die Massage war zwar seit dem Altertum Teil der Heilkunde gewesen, aber Mitte des 19. Jahrhunderts wurde sie eher durch Laienheilkundige verwendet. Professionalisierungsanstrengungen unternahmen v. a. französische und schwedische Ärzte, die Massage und Gymnastik kombinierten. Am bekanntesten ist heute noch die Technik des Fechtlehrers und Dichters Pehr Henrik Ling (1776–1839), der 1813 in Stockholm ein „Gymnastisches Centralinstitut“ gründete und zahlreiche Schüler heranzog [4]. Ab 1842 besuchte der Offizier Märten Thure Emil Brandt (1819–1895, Abb. 1) Kurse am Centralinstitut. 1847 behandelte er den Darmvorfall eines untergebenen Soldaten mit einer kombinierten Therapie: Er massierte mit der rechten Hand die Bauchdecke, während er mit links eine Darmmassage ausführte (Abb. 2; [5]). Ab 1859 begann er auch Frauen zu behandeln, wobei er hier die innere Massage im Genitalbereich ausführte. Er nutzte zeitgenössische ärztliche Techniken, z. B. das gerade erfundene Spekulum zur Abklärung von Unterleibsleiden und er verordnete übergewichtigen Patientinnen eine Diät [6, 7]. Sein wichtigster Eingriff war die „Hebung der Gebärmutter“ bzw. ihre Verlagerung, was seiner Ansicht nach zu einer Verbesserung des Allgemeinbefindens der Patientinnen geführt habe [7]. Damit berührte Brandt eines der zentralen Problemfelder der zeitgenössischen Frauenheilkunde. Die Erkenntnisse der Ärzte fußten auf Beobachtungen an Leichen und hieraus leiteten Gynäkologen nicht nur die Notwendigkeit der Verlagerung der Gebärmutter ab, sondern auch den idealen Sitz des Organs. Um den Uterus zu stabilisieren, setzten die operativ arbeitenden Frauenärzte gemeinhin auf Pessare, Ringe oder gar die Alexander-Adams-Operation zur Vernähung der Gebärmutter mit dem umliegenden Gewebe, was eine Vielzahl von Folgekrankheiten provozierte. In Deutschland galt auf dem Gebiet der Uterusbehandlung der Jenaer Frauenarzt und Universitätsprofessor Bernhard S. Schultze (1827–1919) als führend [8]. Schultzes Name stand aber auch für eine legendäre Fehleinschätzung im Bereich der Genitaldiagnose: 1868 hatte er die erste Transsexuelle der deutschen Medizingeschichte, „Katharina Hohmann“ (1824–1881) untersucht und ihr einen Uterus (in idealer Lage) attestiert, während die Kollegenschaft, darunter Rudolf Virchow (1821–1901), bei Hohmann einen Hoden und somit ein ursprünglich männliches Geschlecht erkannt zu haben glaubten [9]. Am Ende behielt Virchow recht, „Katharina Hohmann“ wanderte in den 1870er-Jahren in die USA aus, heiratete und zeugte als „Karl Hohmann“ mehrere Kinder, was Schultzes akademischem Ansehen nicht unbedingt zuträglich war [10]. Nachdem sich die Kenntnis über die Thure-Brandt-Massage allmählich im deutschsprachigen Raum verbreitet hatte, lud Schultze – unter nicht geringem Erfolgsdruck stehend – den Laien Brandt im Herbst 1886 nach Jena ein, um dort vor Universitätsprofessoren und niedergelassenen Ärzten seine Technik vorzustellen (Abb. 3).

**Figure 1:**
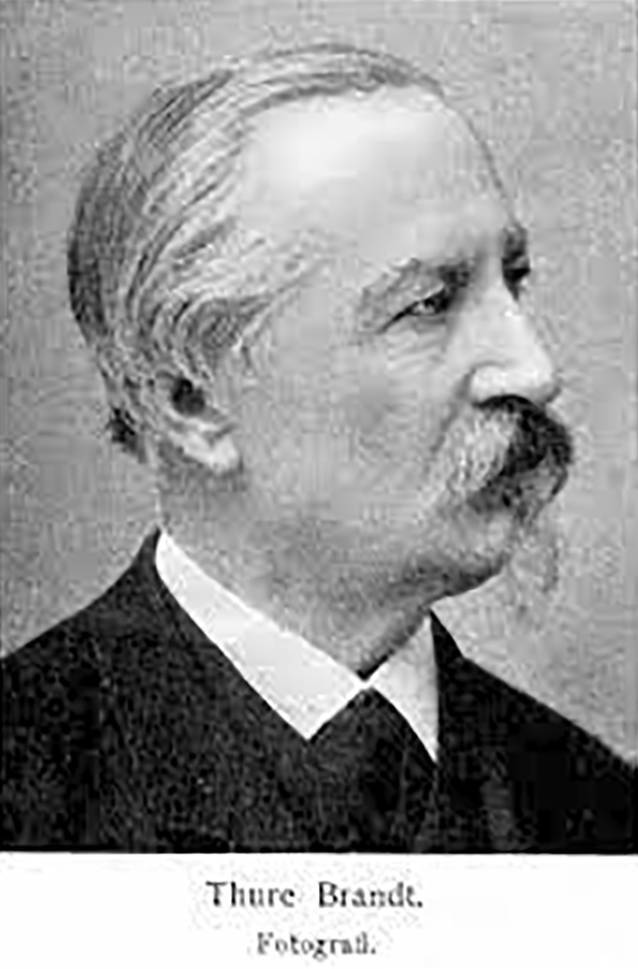

**Figure 2:**
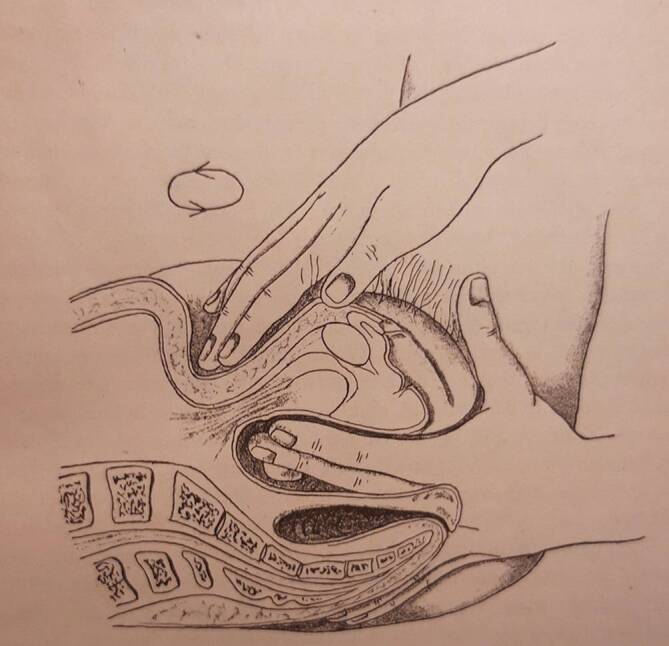

**Figure 3:**
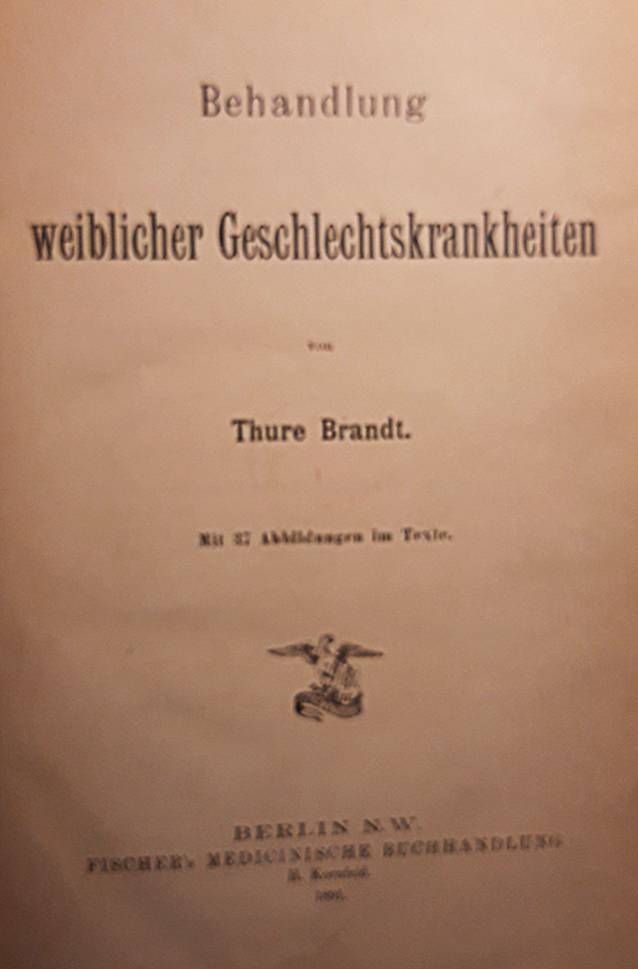

## Präsentation und Problemfelder

Vor Ort gelang es Thure Brandt und dem ihn begleitenden norwegischen Arzt Oskar Nissen (1843–1911), 15 oder 16 von Schultze vorab ausgewählte Patientinnen zu kurieren [11]. Diese Kunde verbreitete sich rasch innerhalb der Ärzteschaft und so erwuchs der Wunsch nach einem Einführungswerk in verständlicher Sprache. Brandt selbst steuerte ein Lehrbuch mit dem missverständlichen Titel „Behandlung weiblicher Geschlechtskrankheiten“ bei, in dem er komplizierte gymnastische Begriffe wie „Lendenkreuz-Beklopfung“ oder „stützstehende“ Behandlung gebrauchte, die deutschen Klinikern weitgehend unvertraut waren [7]. Der Terminus „Geschlechtskrankheiten“ beinhaltete ohnehin eine völlig andere Zielrichtung. Um dem abzuhelfen, präsentierte Brandts eifrigster Befürworter und Anhänger in Deutschland, der an der Münchner Universitätsfrauenklinik tätige Arzt Robert Ziegenspeck (1856–1918, Abb. 4) im Jahre 1895 ein Einführungsbuch, das sich auch an niedergelassene Ärzte wandte (Abb. 5). Darin waren neben zielführenden Abbildungen auch konkrete Hinweise enthalten, was ein noch unerfahrener Arzt im weiblichen Unterleib ertasten würde:

1. „Man nähert die Fingerspitzen dem Ostium vaginae von der Seite, tastet mit dem Daumen nach dem Damme, achtet
2. auf die Beschaffenheit des Introitus, des Hymen, der Vaginalwand während des Eindringens der Finger und des Herabdrängens des Dammes, sucht dann
3. die Spina ischii auf und dreht die Tastfläche der Finger nach vorn, und überzeugt sich von der Stellung der Portio vaginalis zur Spinallinie, wie zur Medianlinie, geht
4. um die Portio herum, überzeugt sich von ihrer Form, sucht
5. in das Ostium einzudringen und erfährt, wie weit es ist und welche Oberfläche die Schleimhaut zeigt, macht
6. den Versuch, die Portio rechts, wie links an die Beckenwand anzudrücken, bzw. auch an das Kreuzbein, bringt
7. die Fingerspitzen der rechten, wie linken Hand leer zusammen, die einen vom Scheidengewölbe aus, die anderen von den Bauchdecken her tastend. Die Hand wird zu erst in der Inguinalgegend aufgelegt. Dann überzeugt man sich
8. ob der Uteruskörper vorn oder hinten oder in Beckenaxe sich befindet, indem man den einen Finger vorn, den anderen hinten an die Zervix anlegt und von den Bauchdecken her gegentastet. Darauf ermittelt man die Form und Größe des Uterus. Hiernach geht man
9. zur Aufsuchung der Ovarien über, ermittelt Größe, Form, Beweglichkeit, wenn möglich auch der Tuben, und erkennt Festigkeit, Dicke und Länge parametritischer Schwielen am besten, indem man sie im angespannten Zustande bimanuell betastet“ (Abb. 2, 6, 7 und 8; [12]).

**Figure 4:**
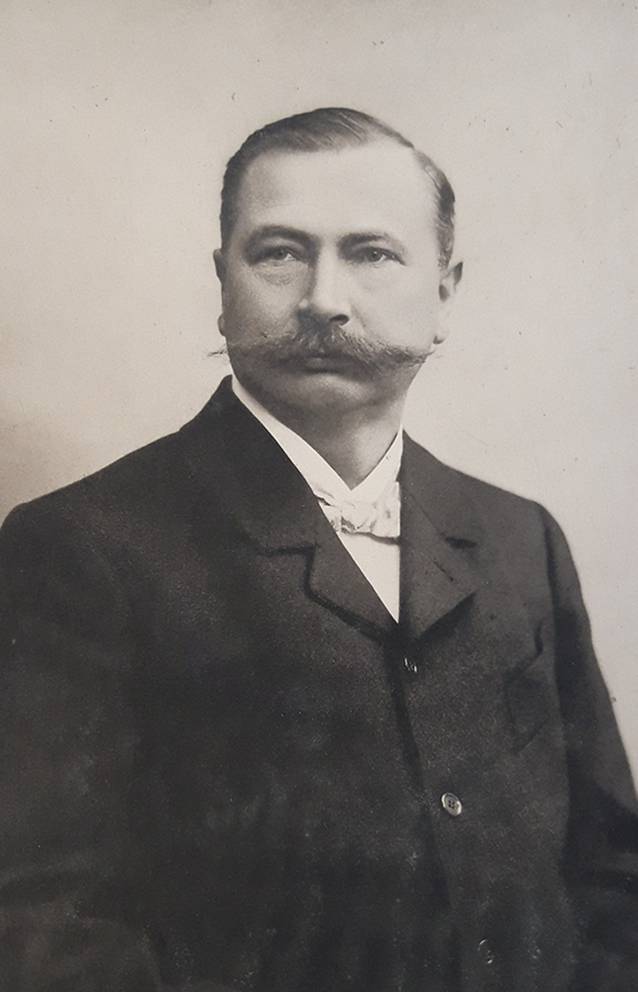

**Figure 5:**
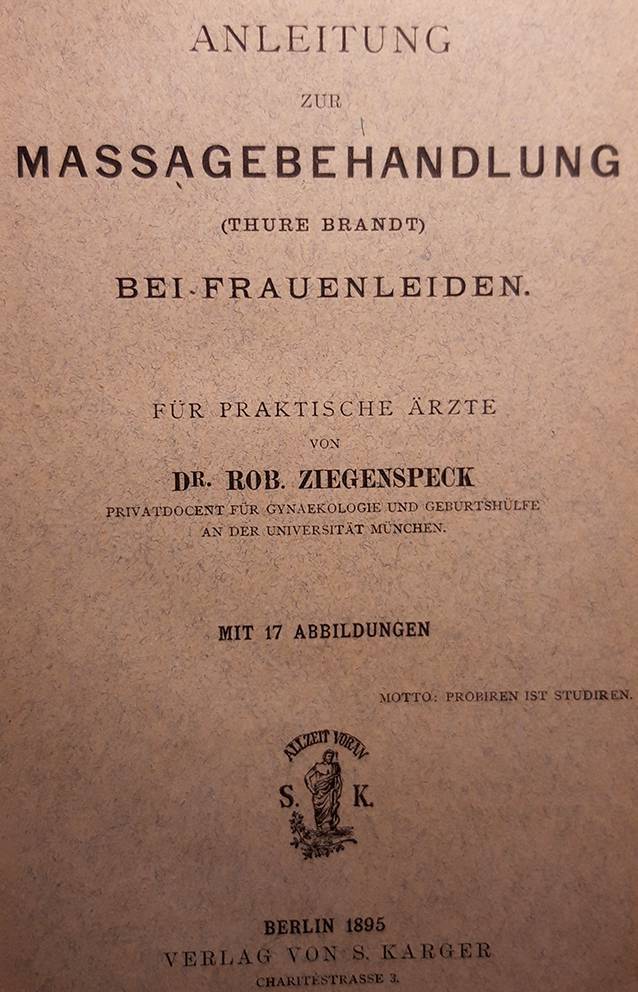

**Figure 6:**
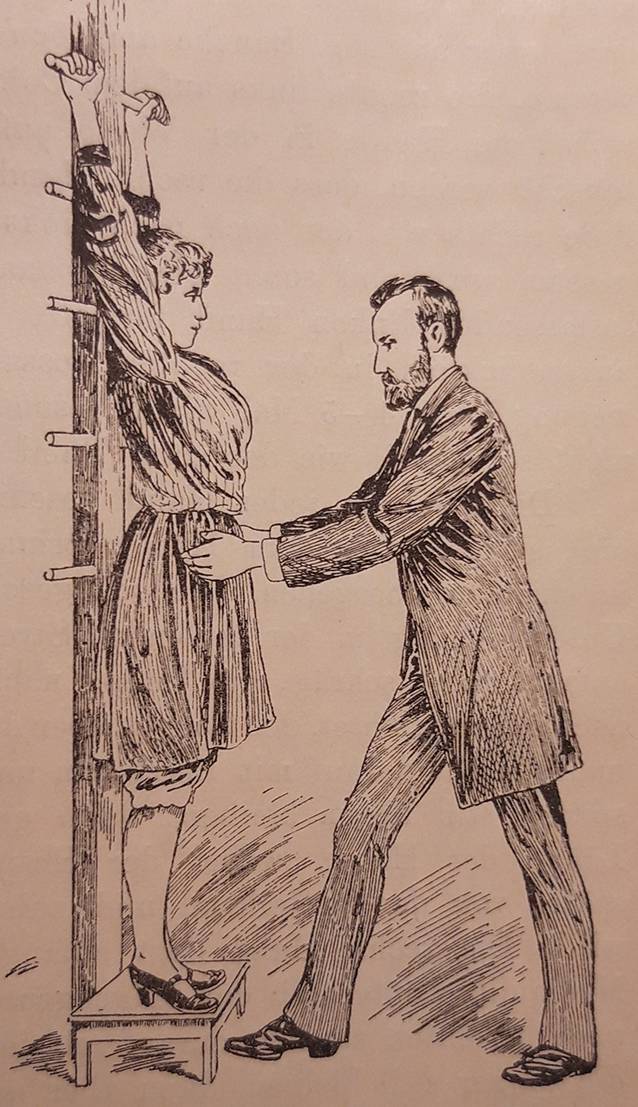

**Figure 7:**
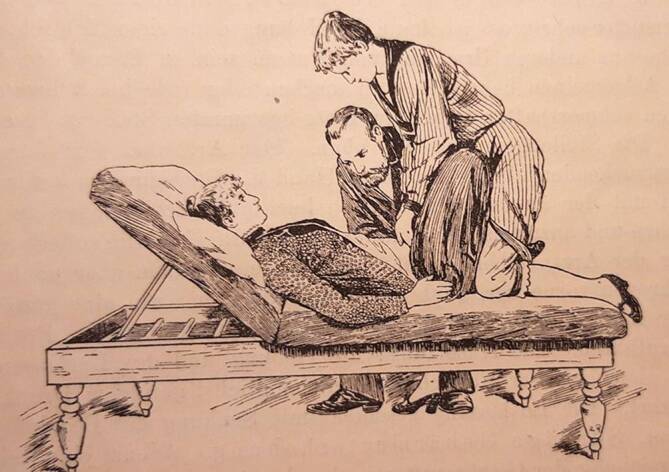

**Figure 8:**
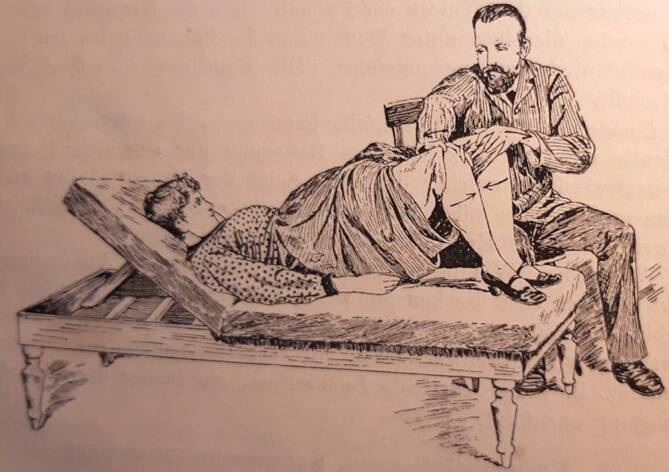

**Figure 9:**
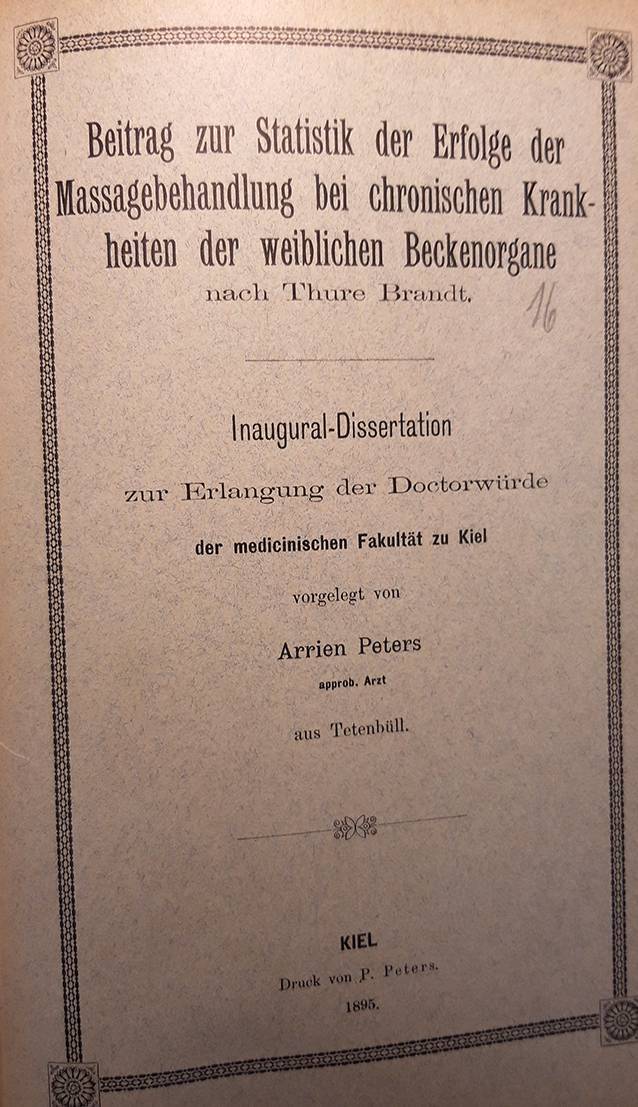

Brandt hatte jedoch seine ersten Behandlungserfolge bei Männern erzielt und dies führte zu entsprechenden Bemühungen niedergelassener Ärzte und Urologen, die Massage bei Prostataleiden in Anwendung zu bringen [13–15]. Hierbei kamen auch Elektrisierapparate zum Einsatz [16]. Entsprechende Behandlungen konnten Sitzungen von bis zu 8 h ununterbrochener Massage von Hand und mithilfe von Sonden beinhalten [17]. Noch zweifelnde Ärzte wurden durch eine klinische Katamnese 1895 überzeugt: Der junge Arzt Arrien Peters (1869–1914) führte an der Universitätsfrauenklinik Kiel eine sich über bis zu 4 Jahre erstreckende Nachuntersuchung von 53 Patientinnen durch, die an verschiedenen Unterleibserkrankungen litten und mit der Thure-Brandt-Massage behandelt wurden [18]. Es zeigte sich, dass im Falle von Gebärmutterverlagerungen, Perimetritis und Sterilität die Mehrheit der Frauen gesundeten (Abb. 9).

Das gesamte Behandlungskonstrukt fußte allerdings, ohne dass dies den Beteiligten klar gewesen wäre, auf zwei fundamentalen Fehleinschätzungen. Nachdem in der zweiten Hälfte der 1890er-Jahre Röntgenuntersuchungen möglich wurden, zeigte sich unter dem Eindruck von Kontrastmitteln zügig, dass die von Schultze und weiten Teilen der deutschen Ärzteschaft geteilte Einschätzung hinsichtlich einer statischen Situation des Uterus falsch war [19]. Der Tübinger Gynäkologe August Mayer (1876–1968) urteilte rückblickend:

„Aufgrund solcher Erfahrungen erscheint das Kapitel Retroflexio uteri manchmal geradezu als ein Trauerspiel in 3 Akten. Der erste beim praktischen Arzt, der die Frauen auf die Knickung ‚einstellt‘ und ihnen damit ein gynäkologisches Krankheitsgefühl ansuggeriert. Der zweite Akt spielt beim Operateur, der durch seine zahlreichen und erfolglosen Eingriffe die Frauen kränker macht, als sie vorher waren. Der dritte Teil spielt beim Psychiater oder Neurologen, der vielleicht von Anfang an die richtige Instanz gewesen wäre“ [20].

Die zweite Fehldiagnose betraf die direkten Wirkungen der Massage, insbesondere der Teil, der innerhalb der Frauenkörper durchgeführt wurde und wesentlich zur Entspannung und der Entstehung von Glücksgefühlen bei den Patientinnen führte. Im Laufe der 1890er-Jahre beschlich eine Reihe von Ärzten der Verdacht, die Therapie sei nichts anderes als Masturbation. Um dem vorzubeugen, wollten die Ärzte die Behandlung normieren und technisieren.

## Sex schafft Probleme

Anstelle ungeschickter Hände sollte eine nur approbierten Akteuren zugängliche Gerätschaft treten – der Vibrator. Die Vibrationsmassage nahm ab 1900/1902 Gestalt an und schien einen unschätzbaren Vorteil zu haben: „Onanistische Reizung ist ausgeschlossen“ [21]. Die Verwendung hochpreisiger technischer Geräte sollte insbesondere dazu dienen, „Streichfrauen und Pfuscher“ von der Behandlung fernzuhalten [22]. Brandt selbst hatte in seinem Erfolgsbuch „Behandlung weiblicher Geschlechtskrankheiten“ auf S. 43 (vgl. Abb. 7) anschaulich dargestellt, wie leicht es sei, Therapeutinnen auszubilden und somit hermetisches, Männern bislang vorbehaltenes ärztliches Wissen unter Laien und insbesondere Frauen zu verbreiten. Die sich nach 1900 entfaltende medizinisch-technische Industrie durchkreuzte allerdings die Pläne der Ärzteschaft ebenso wie die schrittweise Zulassung von Frauen zum Medizinstudium. So blieb letztlich nur ein Streitpunkt um die Thure-Brandt-Massage: ihre möglicherweise schädliche Wirkung im Falle der Schwangerschaft. Brandts wirkmächtiger Epigone Ziegenspeck beteuerte, die Massage sei auch in Zeiten der Gravidität unproblematisch und sogar heilsam [13]. Ziegenspeck blieb ein rühriger Propagandist, veranstaltete Fortbildungsseminare für angehende Mediziner, doch war er wenig geeignet, innerhalb der Welt von Instituts- und Klinikdirektoren als Aushängeschild zu dienen. Denn er hatte gerade zu der Zeit, als er begann, die Brandt-Massage zu bewerben, den einflussreichen Direktor der Münchner Universitätsfrauenklinik Franz v. Winckel (1837–1911) öffentlich als völlig unfähig für diese Position bezeichnet. Er wies nach, dass Winckels Mitarbeiter vorrangig damit beschäftigt waren, die sexuelle Reize der ihnen anvertrauten Patientinnen und Krankenschwestern zu ergründen und sich wenig mit Diagnostik und Therapie aufhielten [23]. Dieser Streit beendete nicht nur Ziegenspecks Karriere an der Universitätsklinik München sondern behinderte auch die Rezeption Brandts. Es zeigte sich zudem, dass weder Gynäkologen noch die an der Staatlichen Massageschule der Charité in Berlin ausgebildeten praktischen Ärzte noch Chirurgen in der Lage waren, die (psycho)somatischen Unterleibsleiden von Frauen und Männern wirksam zu therapieren. Dies begünstigte die Herausbildung der Urologie als neues Spezialfach zur Abwehr von „Pfuschern“. Folgerichtig erwähnten weder Hans Wildbolz (1873–1940) noch Hans Boeminghaus (1893–1979) in ihren einflussreichen Lehrbüchern Thure Brandt [24, 25]. Leopold Casper (1859–1959) hatte sich vage 1910 für die Massage anstelle der Kauterisierung ausgesprochen, ohne jedoch ins Detail zu gehen [26].

Das heilpraktische Angebot stellte aus Sicht der Ärzteschaft ein andauerndes Problem dar [27]. Die Technik wurde spätestens in den 1920er-Jahren von den Naturheilkundigen vollständig okkupiert. In der Rückschau bemerkte der Schweizer Wegbereiter der ärztlichen Naturheilkunde Maximilian Bircher-Benner (1867–1939) kritisch:

„Thure Brandt fand wenige kongeniale Nachfolger unter den Ärzten. Das Messer arbeitet schneller, nur gar oft mit bald vorübergehendem Erfolg. Da und dort wenden Unberufene die Thure-Brandt-Massage an, zum Schaden der Patienten und der Sache“ [28].

**Figure 10:**
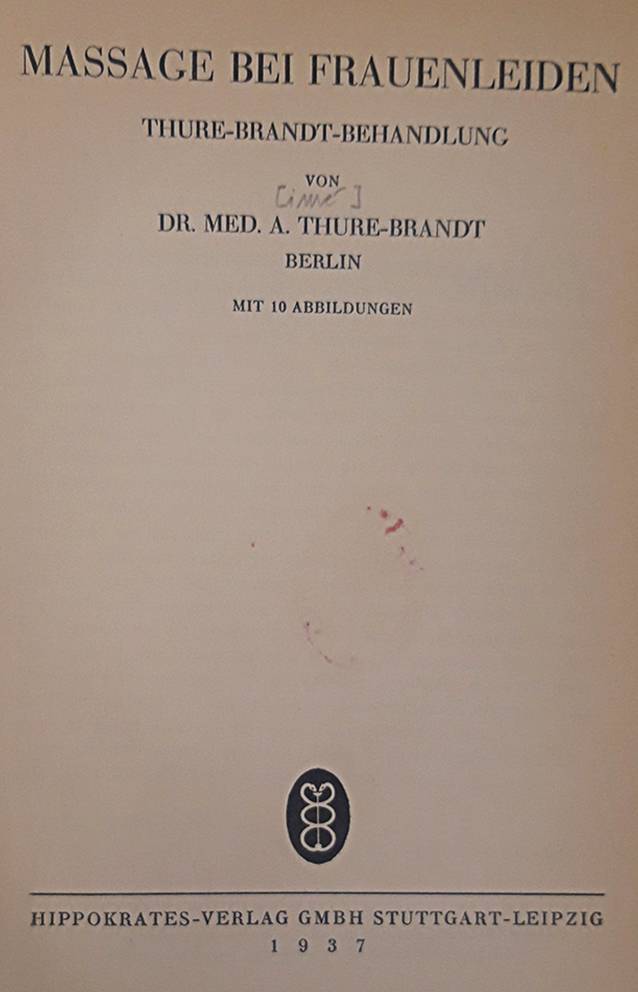

Im Nationalsozialismus kulminierte der Streit um die abortfördernde Wirkung der Massage. Thure Brandts Sohn und Nachfolger Aime Thure Brandt (1857–1946) hatte zwar Medizin studiert und sich somit den formalen Kriterien der Verwissenschaftlichung unterworfen, aber an den Kernaussagen seines Vaters festgehalten. 1937 stellte er ein neues Lehrbuch vor und betonte darin ausdrücklich den Wert der Thure-Brandt-Massage in Zeiten der Schwangerschaft (Abb. 10; [29]). Daraufhin verfasste der Münchner Rechtsmediziner Hermann Merkel (1873–1957) eine grundlegende Kritik der Lehre und bezeichnete die Behandlung als „mechanische Abtreibungshilfe“, die nur von Personen ausgeübt werde, die sich krimineller Methoden der Fruchtabtreibung bedienten [30].

## Nemesis

Mit Merkels Verurteilung von Thure Brandt war die Therapie in der deutschen Ärzteschaft geächtet, aber ganz vorbei war ihre Zeit noch nicht. In der DDR fand sie beschränkt Anhängerschaft und auch der Veteran der ärztlichen Naturheilkunde Alfred Brauchle (1898–1964) bewarb sie in den 1950er-Jahren [31, 32]. Die Sammeldiagnose für ihre Anwendung waren nun „Kreuzschmerzen“ bei Frauen. Versierte Sozialmediziner wussten seit den 1920er-Jahren, dass sich hinter einem solchen Begriff oftmals eine ungewollte Schwangerschaft verbarg. Männer waren als Patientengruppe vollkommen entfallen. Die sexuelle Revolution der 1960er-Jahre und die Verbreitung von Vibratoren entzog die Genitalmassage gänzlich ärztlichen Strukturen. Heute ist Thure Brandt ebenso wie seine Massagetechnik Teil der Medizin- und Kulturgeschichte geworden. Die letzten heilpraktischen Anhänger sollen in den 1970er-Jahren zur Osteopathie gewechselt sein – also gerade zu der Zeit, als die Abtreibung entkriminalisiert wurde. Zur selben Zeit fand „Neotantra“, das in den Kommunen Bhagwans (1931–1990) gelehrt wurde, Eingang in die westliche Esoterik und Alternativkultur. Entsprechende Seminare sind längst Teil der Paartherapie geworden, um den ehelichen Sex wieder in Schwung zu bringen [33].

## Die Thure-Brandt-Massage in der Literatur

Der amerikanische Erfolgsautor Tom Coraghassan („T. C.“) Boyle (eigentl. Thomas John Boyle, geb. 1948) veröffentlichte 1993 den Roman „The Road to Welville“, der im gleichen Jahr in deutscher Sprache unter dem Titel „Willkommen in Wellville“ erschien. Darin schilderte Boyle den therapeutischen Alltag im Jahre 1907 im berühmtesten ärztlich-naturheilkundlichen Sanatorium der USA in Battle Creek in Michigan, das unter der Leitung von John Harvey Kellogg (1852–1943) stand. Kellogg traktierte seine zahlungskräftige Kundschaft mit allen möglichen hydrotherapeutischen und diätetischen Kuren, die neben der Förderung der Gesundheit auf eine Unterdrückung des Sexualtriebs abzielten, um aus dieser Sublimierung kreative Energie abzuleiten. Eine Reihe von Kurgästen empfand dies als Bevormundung und suchte daher den Kontakt zu dem deutschstämmigen „Doktor Spitzvogel“. Dieser „manipuliere den Unterleib“, um so den Körper vor „Autointoxikationen“ zu bewahren und Neurasthenie zu beheben. Ein misstrauischer Ehemann, von Kellogg aufgeschreckt, folgte seiner Ehefrau und ihren Freunden zu einer Blumenwiese unweit von Spitzvogels Praxis:

„Und dann war das sich bewegende Objekt plötzlich deutlich erkennbar, und er sah, daß es ein Mann war, ein Mann, der ihm den Rücken zukehrte, ein nackter Mann, und daß sich sein rechter Arm und seine rechte Schulter rhythmisch auf seine Körpermitte zu bewegten, als ob, als ob … Will war nicht vorbereitet auf das, was er als nächstes sah, hätte es nicht sein können, nicht in seinen wildesten Phantasien, und er erstarrte auf der Stelle.

Er sah, daß dort am Ufer des Flusses zwei Männer und zwei Frauen waren und daß sie nackt waren, alle – vollkommen, gänzlich, total nackt, bis zu den Zehenspitzen. Die Frauen rekelten sich auf dem Rücken, den Kopf an einen Baumstamm gelehnt, und einer der Männer stand zwischen ihnen, seine splitternackten weißen Pobacken der Stelle zugewandt, an der sich Will versteckte. Der Mann hielt seine Arme seitlich ausgestreckt, seine Hände machten sich zwischen den Beinen der Frauen zu schaffen. Der andere Mann – es war Badger – stand direkter hinter ihnen und masturbierte. Und die Frauen? Eine von ihnen, die auf der rechten Seite, war Virginia Cranehill. Ihre großen, sonnengebräunten, schlüpfrigen Zitzen lagen platt auf ihrem Brustkasten, ihre Augen waren geschlossen, der Ausdruck auf ihrem Gesicht ekstatisch. Die andere Frau war Eleanor. Eleanor. Seine Eleanor. Seine Frau. Seine große Liebe. Die sich bewegende Hand hielt sie fest, ihre Brustwarzen waren erigiert, ihre Augen fest geschlossen, und sie stöhnte – stöhnte. Wie ein Tier. Es war ein Bild, das er nicht ertrug. Irgend etwas löste sich, etwas Primitives, Häßliches, und seine Hände suchten zwischen den Blättern nach einer Waffe, der Waffe des Neandertalers, nach einer Keule, einem Knüppel, der harten gemaserten Mordwaffe …“ [34].

Innerhalb weniger Minuten verdrischt der gehörnte Ehemann Spitzvogel und Badger, schnappt sich seine Gattin und reist umgehend aus Battle Creek ab. Eleanor tut alles furchtbar leid, möchte sich erklären („..es war Freikörperkultur, es war Therapie..“), aber Will macht deutlich, sie solle einfach schweigen: „Wir werden nicht darüber sprechen.“ [34]. Jahrzehnte später, nach dem Tod ihres Ehemanns, erinnerte sich Eleanor in den 1950er-Jahren an die Zeit im Sanatorium und bekennt, dass Kellogg in vielem recht behalten habe, „… vielleicht nicht Spitzvogel oder Lionel Badger, der mit neunundvierzig einem Herzschlag erlegen war, und der Gedanke an sie und daran, was zwischen ihnen geschehen war, ließ sie all die vielen Jahre später noch immer erröten und ihr Herz schneller schlagen …“ [34].

Man könnte sagen, Boyle schildert anschaulich die schlimmsten Alpträume der nordamerikanischen und auch deutschen Ärzteschaft hinsichtlich der Thure-Brandt-Massage. Was Anfang des 20. Jahrhunderts skandalös erschien, wäre heute ein gewohnter Anblick im Rahmen von Selbstfindungsseminaren. Wem die Lektüre des 625-seitigen Erfolgsromans zu mühsam erscheint, kann auch zum Film „Wellville“ greifen, der 1994 erschien und in dem Spitzvogels Behandlungsmethode eine wichtige Rolle spielt. 2011 folgte der Streifen „In guten Händen“, in dem der Genitalmassage für Frauen und der Erfindung des Vibrators ein Denkmal gesetzt wurde.

## References

[1] Nöske HD, der Arbeitskreis Geschichte, Urologie (2007). Urologische Balneologie. Urologie in Deutschland. Bilanz und Perspektiven.

[2] Mildenberger FG (2007). Heilende Hände – abtreibende Finger? Die Debatte um die Thure-Brandt-Massge in der deutschsprachigen Medizin (ca. 1870 bis ca. 1970). MedGG.

[3] Hansson N, Ottosson A (2015). Nobel Prize for physical therapy? Rise, fall, and revival of medico-mechanical institutes. Phys Ther.

[4] Schwarzmann-Schafhauser D (2004). Orthopädie im Wandel. Die Herausbildung von Disziplin und Berufsstand in Bund und Kaiserreich (1815–1914).

[5] Resch A (1888). Thure Brandt’s heilgymnastische Behandlung weiblicher Unterleibserkrankheiten.

[6] Brandt T (1886). Die Bewegungscur als Heilmittel gegen weibliche sog. Unterleibsleiden und Prolapsen. Anzeichnungen seit dem Jahre 1861.

[7] Brandt T (1891). Behandlung weiblicher Geschlechtskrankheiten.

[8] Schultze BS (1881). Pathologie und Therapie der Lageveränderungen der Gebärmutter.

[9] Virchow R (1872). Vorstellung eines Hermaphroditen. Berl Klin Wochenschr.

[10] Mak G (2011). Hermaphrodites on Show. The case of Katharina/Karl Hohmann and its use in nineteenth-century Medical Science. Soc Hist Med.

[11] Heyll U (2006). Wasser, Fasten, Luft und Licht. Die Geschichte der Naturheilkunde in Deutschland.

[12] Ziegenspeck R (1895). Anleitung zur Massagebehandlung (Thure Brandt) bei Frauenleiden. Für praktische Ärzte.

[13] Ebermann AL (1892). Die Massage der Prostata. Int Cbl Physiol Pathol Harn Sex Org.

[14] Rosenberg S (1894). Die Therapie der Prostatitis chronica. Int Cbl Physiol Pathol Harn Sex Org.

[15] Feleki H (1895). Beiträge zur Kenntnis und Therapie der chronischen Entzündung der Prostata und der Samenbläschen. Int Cbl Physiol Pathol Harn Sex Org.

[16] Moll FH, Löffelbein N, Halling T, Fangerau H (2020). Die Urologie wird elektrisch – Elektrotherapie. Moderne Therapien zur Behandlung moderner Erkrankungen – Elektrotherapie. Der Urologe.

[17] Hohnbaum A (1906). Ueber Vibrationsmassage.

[18] Peters A (1895). Beitrag zur Statistik der Erfolge der Massagebehandlung bei chronischen Krankheiten der weiblichen Beckenorgane nach Thure Brandt.

[19] Hölder H (1912). Zur Kritik der Retroflexio uteri mobilis.

[20] Mayer A (1939). Bedeutung und Behandlung der Retroflexio uteri. Geburtshilfe Frauenheilkd.

[21] Witthauer K (1905). Lehrbuch der Vibrationsmassage mit besonderer Berücksichtigung der Gynäkologie.

[22] Reibmayr A (1883). Die Massage-Behandlung. Populär dargestellt.

[23] Mildenberger FG (2009). Robert Ziegenspeck (1856–1918) Der „Don Quichotte“ der ambulanten Gynäkologie. Nachtrag zum Aufsatz über Thure Brandt. MedGG.

[24] Wildbolz H (1924). Lehrbuch der Urologie und chirurgischen Krankheiten der männlichen Geschlechtsorgane.

[25] Boeminghaus H (1927). Urologische Diagnostik und Therapie für Ärzte und Studierende.

[26] Casper L (1910). Lehrbuch der Urologie mit Einschluss der männlichen Geschlechtskrankheiten.

[27] Kirchberg F (1930). Massage und Gymnastik in Schwangerschaft und Wochenbett.

[28] Bircher-Benner M (1938). Vom Werden des neuen Arztes. Erkenntnisse und Bekenntnisse.

[29] Brandt AT (1937). Massage bei Frauenleiden. Thure-Brandt-Behandlung.

[30] Merkel H (1940). Der gerichtsärztliche und kriminalistische Nachweis der Abtreibung. Dtsch Z ges gerichtl Med.

[31] Grüger A (1956). Parametritis posterior als Ursache für die Kreuzschmerzen der Frau.

[32] Brauchle A (1957). Das große Buch der Naturheilkunde.

[33] Koppetsch C, Speck S (2015). Wenn der Mann kein Ernährer mehr ist. Geschlechterkonflikte in Krisenzeiten.

[34] Boyle TC (2014). Willkommen in Wellville.

[35] Ziegenspeck R, Volkmann R (1890). Über Thure Brandt’s Verfahren der Behandlung von Frauenleiden. Sammlung klinischer Vorträge in Verbindung mit deutschen Klinikern.

